# Clinical Severity and Calcium Metabolism in Patients with Bipolar Disorder

**DOI:** 10.3390/brainsci10070417

**Published:** 2020-07-01

**Authors:** Luca Steardo, Mario Luciano, Gaia Sampogna, Elvira Anna Carbone, Vito Caivano, Arcangelo Di Cerbo, Vincenzo Giallonardo, Carmela Palummo, Alfonso Vece, Valeria Del Vecchio, Pasquale De Fazio, Andrea Fiorillo

**Affiliations:** 1 Department of Psychiatry, University of Campania “Luigi Vanvitelli”, 80131 Naples, Italy; mario.luciano@unicampania.it (M.L.); gaia.sampogna@unicampania.it (G.S.); vito.caivano@unicampania.it (V.C.); ardice77@gmail.com (A.D.C.); enzogiallo86@gmail.com (V.G.); carmela_palummo@libero.it (C.P.); alfonsovece90@gmail.com (A.V.); valeria.delvecchio78@gmail.com (V.D.V.); andrea.fiorillo@unicampania.it (A.F.); 2 Psychiatric Unit, Department of Health Sciences, University Magna Graecia, 88100 Catanzaro, Italy; elvira.carbone@libero.it (E.A.C.); defazio@unicz.it (P.D.F.)

**Keywords:** bipolar disorder, calcium homeostasis, parathyroid hormone, vitamin D, severity, long-term outcome

## Abstract

Parathyroid hormone (PTH), vitamin D and serum calcium play a key role in several physiological and pathological conditions. Vitamin D and PTH receptors are largely expressed in the central nervous system and are involved in the modulation of inflammatory responses. Few studies investigated the association between calcium homeostasis imbalance and psychiatric disorders. This study aims to assess calcium homeostasis imbalance in patients with bipolar disorder (BD) and its impact on clinical outcome. We recruited 199 patients with BD, who were administered with validated assessment instruments to investigate depressive, manic and anxiety symptoms, affective temperaments, childhood trauma and global functioning. Serum calcium, vitamin D and PTH levels were assessed in all patients. Levels of PTH correlated with several clinical characteristics, including the diagnosis of bipolar disorder type I (BD-I), the presence of psychotic symptoms, lithium treatment, suicidality, total number of acute episodes and of hospitalizations (*p* < 0.0001) and seasonality (*p* < 0.05). At the regression analyses, higher levels of PTH were predicted by early age at onset, number of hospitalizations, aggressive behaviors (*p* < 0.05), higher Childhood Trauma Questionnaire total score (CTQ) (*p* < 0.001) and treatment with lithium (*p* = 0.01). Our findings suggest that the calcium homeostasis could play a role in BD patients, and that PTH levels are correlated with the clinical severity of the disorder.

## 1. Introduction

Calcium homeostasis is implicated in several physiologic processes, such as the homeostasis balance of the muscle skeletal system, the immune modulation, the antioxidant defense system and in several inflammatory processes [1]. The homeostasis of calcium is largely regulated by an integrated hormonal system that modulates calcium transportation in the gut, kidney and bone, through the involvement of parathyroid hormone (PTH) [2], vitamin D (Vit D), serum-ionized calcium and calcium-sensing receptor (CaR) [3]. Vitamin D stimulates brain cells to produce several growth factors, like nerve growth factor (NGF), glial cell line-derived neurotrophic factor (GDNF) and neurotrophin 3 (NT3), thus stimulating the protection and growth of neuronal cells. The Vit D receptor (VDR), which is largely expressed in the central nervous system (CNS), especially in the amygdala, regulates behavioral and emotional responses [4] and is involved in modulating inflammatory responses. Its ligand, Vit D, is a membrane antioxidant which increases gene expression of several antioxidant agents [5,6]. Vit D also decreases cytokines activity via inhibitory effects on the activation and expression of inflammatory factors such as interleukins 1 and 6, Tumor necrosis factor-α (TNFα), nuclear factor kappa B (NF-κB) and other related genes [7]. 

Moreover, evidence has suggested that Vit D modulates the biosynthesis of neurotransmitters, such as serotonin through tryptophan hydroxylase 2 (TPH2), and neurotrophic factors, thus significantly influencing mood and its alterations [8,9].

Moreover, the PTH regulates the levels of circulating and intracellular calcium in the CNS, inducing apoptosis due to calcium overloading [10]. Elevated PTH levels are associated with a reduced regional cerebral blood flow [11], whereas PTH-related protein (PTHrP) inhibits the activity of the calcium channel, thus contributing to maintaining normal neuronal function [12]. PTH also promotes the conversion of Vit D to its active form [13], being involved in neuroprotective and anti-inflammatory regulation [14]. High levels of PTH could be associated to neural damages. In fact, a higher calcium concentration can be found in the CNS because of PTH imbalance. Calcium overload can lead to neuronal signaling disruption or atrophy in the hippocampus, with the onset of psychiatric symptoms [15].

Several studies suggest that decreased blood levels of Vit D are involved in dementia, Parkinson’s disease and psychiatric disorders, in particular, affective disorders [16,17]. In fact, Vit D deficiency seems to be strongly correlated with the severity of depressive symptoms, and a recent metanalysis reported the beneficial effects of vitamin D supplementation in patients with depression [18]. As regards bipolar disorder (BD), a significant association between vitamin D deficiency and severity of illness has been found. This might be due to different factors. Calcium metabolism is directly involved in serotonin biosynthesis and is correlated to mood swings and impulsive behaviors [19]; moreover, a decrease of neuronal plasticity has been found in different brain regions involved in bipolar disorder consequent to Vit D decrease [20]. Lastly, the generalized and chronic inflammation associated to calcium imbalance sustain an activation of the hypothalamic-pituitary-axis, with consequent mood alteration [21]. Therefore, calcium, Vit D and PTH levels can be used as a marker of chronic inflammation and, consequently, of chronic neuroinflammation [21]. In fact, patients with an acute manic episode present lower serum concentrations of Vit D, compared with healthy controls and patients in remission [22]. More recently, it has been suggested that, compared to healthy controls and psychotic disorders, the acute phase of BD is associated with a rise in plasma Vit D synthesis and with a low-grade inflammation [23].

Despite the fact that several studies have showed the link between calcium metabolism and mood fluctuations, most of these studies have focused mainly on the effect of Vit D on depressive symptoms, while only a few studies have explored the effect of the alterations of the whole calcium homeostasis (including the levels of PTH and calcium) on the course of patients with bipolar disorder. PTH can be considered a more accurate proxy of chronic calcium homeostasis imbalance.

In this study, we assessed calcium homeostasis imbalance in a sample of patients with bipolar disorder (BD); in particular, we explored whether serum levels of PTH, Vit D and calcium influence the clinical presentation of bipolar disorder, its symptom severity and clinical outcome.

## 2. Methods

### 2.1. Participants

This is an observational naturalistic study. Patients were consecutively recruited at the Psychiatric Units of the University of Campania “Luigi Vanvitelli” in Naples and of the University Hospital Mater Domini in Catanzaro, from June 2019 to March 2020. Patients were included in the study if they met the following criteria: (1) age between 18 and 65 years, (2) diagnosis of type-I or type-II bipolar disorder according to the Diagnostic and Statistical Manual of Mental Disorders—fifth edition (DSM-5) [24] and (3) willingness to participate in the study. Patients were excluded in case of: (1) inability to give written consent to participate in the study, (2) moderate or severe cognitive impairment, (3) comorbidity with any neurologic disease or drug and/or alcohol abuse, (4) being pregnant or in the post-partum period and (5) currently in treatment with medications that can alter the calcium metabolism. All patients gave their written informed consent to participate in the study after receiving a full description of the study aims and design. The study was carried out in accordance with the latest version of the Declaration of Helsinki and was approved by the Ethics Committee of the University of Campania “Luigi Vanvitelli” (number: N001567/28.01.2018.).

### 2.2. Procedures and Measures

#### 2.2.1. Socio-demographic and Clinical Characteristics

Patients’ clinical and socio-demographic characteristics, including gender, age at study entry, employment status, educational level, family history of psychiatric illnesses, type of onset, lifetime number of affective episodes, pattern of illness course, treatments, suicidal ideation and previous psychiatric hospitalizations, were recorded with an ad-hoc schedule.

The severity of depressive and anxious symptoms was assessed with the Hamilton Depression Rating Scale (HAM-D) [25] and the Hamilton Rating Scale for Anxiety (HAM-A) [26], manic symptoms were assessed with the Mania Rating Scale [27] and affective temperaments were explored with the short Italian version of the Temperament Evaluation of Memphis, Pisa, Paris and San Diego ( brief TEMPS-M) [28]. The presence of emotional, sexual or physical childhood abuse and of emotional or physical neglect was investigated through the Childhood Trauma Questionnaire—Short Form (CTQ-SF) [29].

#### 2.2.2. Assessment of Serum Parameters

In order to determinate the serum levels of calcium, 25-OH-vitamin D and PTH, biological samples were obtained from all patients and were assessed at recruitment in the laboratories of the two participating sites, adopting a standardized procedure. Calcium was measured using standard laboratory methods. Blood was centrifuged and serum was stocked at −30 ˚C for 25-OH-vitamin D and PTH and evaluated by chemiluminescence immunoassays using adequate kits (Diasorin Liaison; ADVIA Centaur).

#### 2.2.3. Statistical Analyses

Descriptive statistics were calculated for socio-demographic and clinical characteristics as well as for other relevant assessment instruments. Data are presented as means and standard deviations (SD) or frequencies and percentages (%), as appropriate. The Kolmogorov–Smirnov test was adopted to check the normality of distribution of our sample. Correlation analyses have been performed to explore the association of serum levels of PTH, Vit D and calcium with continuous variables. The Student’s t-test for independent samples was performed to assess the association of serum levels of PTH, Vit D and calcium with discrete variables. Linear regression analyses were performed using PTH, Vit D and calcium as dependent variables, and independent variables were selected among those with a positive association at the univariate analyses and among those identified as most relevant from the literature [30]. The level of statistical significance was set at *p* < 0.05. Statistical analyses were performed with the Statistical Package for Social Sciences, version 21 (SPSS, Chicago, IL, USA).

## 3. Results

### 3.1. Socio-Demographic Characteristics of the Sample

The total sample consisted of 199 patients, 98 being males (49%) and 101 females (51%), with a mean age of 47.1 ± 13.2 years. Half of them were in a stable relationship (53%) and employed (55%). Seventy percent of the sample had a positive history for psychiatric disorders, and 109 subjects (55%) had received a diagnosis of BD-I. The mean age at onset was 27.0 ± 9.5, and the duration of illness was 20 years (±12.4). Half of the sample (51%) was in treatment with lithium. Blood levels were 42.6 ± 21.6 pmol/L for PTH, 42.6 ± 65.3 ng/mL for Vit D and 9.4 ± 0.8 mg/mL for calcium. The main socio-demographic and clinical characteristics of the sample are reported in Table 1.

### 3.2. Univariate Analyses

Correlations between serum parameters and clinical variables are reported in Table 2 and Table 3. The levels of serum PTH were inversely correlated with age at onset (*p* < 0.01), years of education (*p* < 0.05) and age at first depressive episode (*p* < 0.01); on the contrary, they were directly correlated with the total number of hospitalizations (*p* < 0.01), and of depressive (*p* < 0.0001), manic (*p* < 0.001) and hypomanic episodes (*p* < 0.01). Higher levels of PTH have been found in patients with a diagnosis of BD-I (*p* < 0.0001), regular course of illness (*p* = 0.028), aggressive behaviors (*p* = 0.009), psychotic symptoms during depressive (*p* < 0.0001) or maniac episodes (*p* < 0.0001), seasonal pattern of the illness (*p* = 0.023), use of atypical antipsychotics (*p* = 0.013) and a history of suicide attempts (*p* < 0.0001). Moreover, the PTH was positively correlated with the depressive (*p* < 0.0001), anxious (*p* < 0.01), cyclothymic (*p* < 0.001) and irritable (*p* < 0.0001) temperaments at the brief TEMPS-M. Emotional neglect, emotional abuse, physical abuse, physical neglect, trauma and CTQ total score were also strongly correlated (*p* < 0.0001) with elevated PTH levels. No correlation was found with gender, educational and occupational levels, psychiatric history, use of drugs, prevalent polarity (both depressive or manic) during illness, treatment with anticonvulsants, typical antipsychotics or selective serotonin reuptake inhibitors (SSRI) antidepressants.

Serum levels of calcium were positively correlated with years of education and with the HAM-A total score (*p* < 0.05). Serum levels of Vit D were positively associated with age at first psychiatric contact and were inversely correlated with the total number of depressive episodes (*p* < 0.05) and cyclothymic temperament (*p* < 0.05). Moreover, higher levels of Vit D were reported in patients without psychotic symptoms during the acute phases of the disorder (*p* < 0.05).

### 3.3. Multivariate Analyses

The results of the regression linear analysis are reported in Table 4. The model was run to assess the independent predictors associated with the PTH levels. Patients with higher levels of PTH are more likely to have a younger age of onset (*p* = 0.032), higher number of hospitalizations (*p* = 0.017), aggressive behaviors (0.092), higher score at the CTQ scale (*p* = 0.001) and to be in treatment with lithium (*p* = 0.013).

## 4. Discussion

The main finding of our study is the association of high PTH levels with the severity of illness (i.e., highly recurrent patients with a positive history of childhood trauma and impulsivity), which can be due to several factors. First, PTH levels can influence the levels of chronic neuroinflammation, which is significantly associated with a higher burden of illness and a more severe clinical presentation of the disorder [31], leading to an altered neurotransmission [32] and dysfunctional brain development through a reduced induction of the nerve growth factor [33,34], reduced immunoregulation and anti-inflammatory actions [35,36]. Second, the relationship between high PTH levels and age at onset might be due to a chronic Vit D deficiency, that triggers the neuroinflammation process, ultimately resulting in full-blown disease [37,38]. This finding confirms previous evidence that calcium imbalance is associated with an earlier age at onset and with a worse long-term outcome in terms of symptom severity, social functioning and number of relapses and hospitalizations [39]. In our study, age at onset and number of hospitalizations both predict higher PTH levels, thus confirming the role of PTH in worsening the long-term outcome of BD. Third, the association between aggressive behaviors and high levels of PTH can be explained by the role of calcium imbalance in the synthesis of serotonin neurotransmitters through the tryptophan pathways [40]. In fact, the involvement of serotonin in the regulation of impulsivity and its clinical correlates turns into reduced brain serotonin levels, which cause antisocial/aggressive behaviors, feelings of anger and self-injuries [41,42,43,44]. In BD, the alteration of the serotonin synthesis is associated with aggressive behaviors [40,45] and a higher risk of suicide attempts [46]. However, we did not assess serotonin levels in our sample, and therefore, this explanation deserves further studies to be confirmed. Fourth, the CTQ total score is also associated with higher PTH levels. Childhood traumas increase the lifetime risk to develop BD [47] and are associated with a greater clinical severity [48] and a more severe clinical course (i.e., rapid cycling, early age of onset, suicide attempts and more depressive episodes) [49]. In BD patients, a history of childhood trauma leads to difficulties in affective regulation, impulse control, cognitive functioning and neurobiological modifications due to the epigenetic consequences of early trauma. The hypothalamic-pituitary-adrenal (HPA) axis, serotonergic transmission, inflammation, neuroplasticity and calcium signaling are all involved in these modifications [50]. It is possible that the levels of PTH, Vit D and calcium are influenced by traumatic events, as it happens with the HPA axis; despite this, the explanation deserves further investigation. Lastly, according to our regression model, higher levels of PTH are predicted by a pharmacological treatment with lithium. This result is in line with available studies, which have found that a long-term treatment with lithium stimulates the parathyroid function, leading to a secondary hyperparathyroidism [51]. Although the real prevalence of this phenomenon is unclear, about 25% of lithium-treated patients have disturbances in the calcium homeostasis [52]. Lithium interacts with the calcium-sensing receptor and causes intracellular changes in calcium levels in parathyroid chief cells, thereby influencing PTH secretion [53].

According to the univariate analyses, several severity and outcome indexes, including the diagnosis of bipolar I disorder, the presence of psychotic characteristics in acute phases, seasonality and history of suicide attempts, are associated with increased levels of PTH. All these indexes are correlated with a worse outcome and a higher psychological distress in BD [54,55,56,57]. An evident, but less significant relationship was found between the levels of calcium and Vit D with symptom severity. This result, which is in line with previous studies [58], confirms the role of calcium metabolism in mood stability, with a worse outcome and a greater severity of the illness in patients with a calcium metabolism imbalance.

Differently from what was reported in other studies, in our sample, the clinical outcome variables were more frequently correlated with the serum levels of PTH rather than with those of Vit D. This can be due to the fact that in our sample, the serum levels of Vit D and calcium were within normal ranges (mean Vit D levels in our sample: 42.57 ± 65.31 ng/mL, normal ranges: 30–100 ng/mL, mean serum-ionized calcium in our sample: 9.42 ± 0.76 mg/dl, normal ranges: 9–10.7 mg/dl), while the PTH levels were higher than normal (mean PTH levels in our sample: 45.6 pmol/L, normal ranges: 7–10 pmol/L). This finding, which is indicative of a secondary hyperparathyroidism, confirms the presence of a chronic imbalance in calcium metabolism in BD patients. Moreover, differently from PTH, the levels of Vit D and calcium are also influenced by other external variables (i.e., diet, Ultraviolet UV light, physical exercise), and therefore, represent a less accurate expression of a chronic calcium metabolism imbalance [59].

To our knowledge, this is one of the few available studies which evaluated the association between increased levels of PTH and clinical severity and outcome in patients with BD. Its main strengths include the concomitant assessment of PTH, Vit D and calcium levels, which allows the analysis of the whole metabolism axis and the relatively high sample size, compared with other available studies. However, the study also has some important limitations, one being the absence of a control group. Moreover, the cross-sectional design of the study limits conclusions about causality. A longitudinal evaluation of serum parameters will allow us to clarify variability of PTH, calcium and Vit D levels according to the psychopathological variables. Another possible limitation is the absence of a retrospective assessment of serum parameters, especially the levels at the onset of illness and during its acute phases. However, we have planned follow-up assessments in order to counterbalance this limitation.

In conclusion, increased levels of PTH correlate with a worse outcome and a high psychological burden in BD patients. Our results suggest that calcium imbalance may influence the long-term outcome of bipolar disorder and highlight the importance to routinely assess PTH, Vit D and calcium levels in these patients as a marker of clinical severity.

## Figures and Tables

**Table 1:** Socio-demographic and clinical characteristic of total sample.

	Sample (*N* = 199)	
Socio-demographic Characteristics	Age, M (±SD)	47.1 (±13.2)
	Gender, Male, N (%)	98 (49.2)
	Years of Education, M (±SD)	13.3 (± 3.5)
	Having Partner, Yes, N (%)	52.8 (103)
	Employed, Yes, N (%)	55.4 (107)
	Diagnosis of BD-I, N (%)	54.8 (108)
	Family History of Psychiatric Disorder, Yes N (%)	69.8 (88)
Clinical Variables	Age of Onset, M (±SD)	26.9 (±9.5)
	Age First Psychiatric Contact, M (±SD)	29.8 (±9.9)
	Age First Depressive Episode, M (±SD)	27.7 (±9.1)
	Age First Manic Episode, M (±SD)	29.6 (±8.4)
	Age First Hypomanic Episode, M (±SD)	30.2 (±8.7)
	Age First Mixed Episode, M (±SD)	33.9 (±10.0)
	Total Number of Depressive Episodes, M (±SD)	5.7 (±6.0)
	Total Number of Manic Episodes, M (±SD)	3.6 (±3.2)
	Total Number of Hypomanic Episodes, M (±SD)	3.1 (±3.2)
	Total Number of Episodes, M (±SD)	10.9 (±10.4)
	Total Number of Episodes during Last Year M (±SD)	0.9 (±0.9)
	Prevalent Polarity, Yes, N (%)	26.5 (49)
	Suicide Attempts, Yes, N (%)	30.2 (60)
	Number of Suicide Attempts, M (±SD)	0.4 (±0.9)
	Aggressive Behaviors, Yes, N (%)	56.8 (112)
	Psychotic Symptoms, Yes, N (%)	42.3 (93)
	Psychotic Symptoms during Depressive Episode, Yes, N (%)	28.6 (57)
	Psychotic Symptoms during Manic Episode, Yes, N (%)	46.0 (92)
	Seasonality, Yes, N (%)	44.9 (89)
	Treatment with Lithium, Yes, N (%)	52.8 (105)
	Total Number of Hospitalizations, M (±SD)	0.7 (±0.9)
	Illness Duration, M (±SD)	20.0 (±12.4)
	Untreated Illness Duration, M (±SD)	2.8 (±5.6)
	Course of Illness, Regular, N (%)	63.5 (116)
	Previous Se of Drugs, yes, N (%)	25.6 (51)
Serum Variables, M (±SD)	PTH (pmol/L)	45.6 (±21.6)
	Calcium (mg/dL)	9.42 (±0.76)
	25-OH-vitamin D (ng/mL)	42.57 (±65.31)
B-TEMPS, M (±SD)	Depressive	22.6 (±6.7)
	Hyperthymic	19.1 (±6.1)
	Anxious	18.9 (±6.2)
	Cyclothymic	23.3 (±7.6)
	Irritable	18.9 (±8.1)
HAM-A, M (±SD)	Total Score	4.7 (±7.2)
HAM-D, M (±SD)	Total Score	7.9 (±10.8)
MRS, M (±SD)	Total Score	4.6 (±8.4)

BD-I: type I bipolar disorder; PTH: parathyroid hormone; B-TEMPS: Temperament Evaluation of Memphis, Pisa, Paris and San Diego; HAM-A: Hamilton Rating Scale for Anxiety; HAM-D: Hamilton Depression Rating Scale; MRS: Mania Rating Scale; M: mean; SD: Standard deviation

**Table 2:** Student’s t-test comparing clinical characteristics and calcium metabolism.

	Variables		Total Sample *N* = 199	Mean	Standard Deviation	*p*
PTH	Diagnosis	BD-I	108	52.26	22.02	0.000
		BD-II	91	36.87	17.85	
	Course of Illness	Regular	116	48.90	21.45	0.028
		Irregular	83	40.15	21.06	
	Aggressive Behaviors	No	87	39.44	21.07	0.009
		Yes	112	49.72	21.17	
	Psychotic Symptoms	No	105	35.40	18.15	0.000
		Yes	93	58.20	19.04	
	Psychotic Symptoms during Depressive Episode	No	142	42.72	20.55	0.000
		Yes	57	63.16	19.59	
	Psychotic Symptoms during Maniac Episode	No	107	36.60	18.99	0.000
		Yes	92	57.51	19.03	
	Seasonality	No	106	41.79	21.01	0.023
		Yes	93	50.67	21.54	
	Treatment with Lithium	No	94	33.93	17.95	0.000
		Yes	105	55.08	19.57	
	Treatment with Atypical Antipsychotics	No	61	35.79	19.65	0.013
		Yes	138	47.65	21.43	
	Suicide Attempts	No	128	39.62	19.88	0.000
		Yes	71	61.63	17.87	
Calcium*	High School Degree	No	54	9.06	1.70	0.030
		Yes	145	9.48	0.45	
	Prevalent Manic Polarity	No	152	9.40	0.79	0.049
		Yes	47	9.65	0.32	
25-OH-Vitamin D*	Psychotic Symptoms	No	115	36.74	12.90	0.046
		Yes	84	32.16	11.13	
	Psychotic Symptoms during Depressive Episode	No	140	35.62	12.46	0.043
		Yes	59	29.33	10.20	

BD-I: type I bipolar disorder; BD-II: type II bipolar disorder. *Correlations among Calcium, 25-OH-vitamin D and other socio-demographic and clinical characteristics are not statistically significant and have not been reported.

**Table 3:** Pearson rho correlations of clinical variables and psychopathological scores.

Variables		PTH	Calcium	25-OH-Vitamin D
PTH		1	0.000	−0.260 ***
Calcium		0.000	1	−0.179
25-OH-Vitamin D		−0.260 ***	−0.179	1
Age		0.006	−0.030	0.107
Education (years)		−0.172 *	0.181 *	0.002
Age of Onset		−0.221**	0.037	0.128
Age First Psychiatric Contact		−0.155	0.009	0.179 *
Untreated Illness Duration		0.139	−0.061	0.113
Age First Depressive Episode		−0.219 **	0.000	0.145
Age First Manic Episode		−0.080	0.012	0.041
Age First Hypomanic Episode		−0.143	−0.051	0.145
Total Number of Hospitalizations		−0.405 ****	−0.052	−0.078
Illness Duration		0.173	−0.060	0.017
Total Number of Depressive Episodes		0.411 ****	−0.020	−0.182 *
Total Number of Manic Episodes		0.357 ***	0.042	0.012
Total Number of Hypomanic Episodes		0.226 **	0.022	−0.074
Total Number of Mixed Episodes		−0.231	0.080	−0.015
Total Number of Episodes		0.442 ****	−0.009	−0.143
Total Number of Episodes during Last Year		0.114	0.048	−0.143
Number of Suicide Attempts		0.399 ****	0.011	−0.033
TEMPS-B	Depressive	0.319 ****	−0.054	0.015
	Hyperthymic	−0.085	−0.029	−0.084
	Anxious	0.215 **	−0.128	0.016
	Cyclothymic	0.242 ***	−0.077	−0.185 *
	Irritable	0.417 ****	0.160	−0.067
CTQ	Emotional Neglect	0.575 ****	0.107	−0.157
	Emotional Abuse	0.590 ****	0.015	−0.028
	Sexual Abuse	−0.104	−0.071	−0.002
	Physical Neglect	0.586 ****	0.0999	−0.137
	Physical Abuse	0.578 ****	0.115	−0.125
	Trauma	0.588 ****	0.061	−0.244 *
	CTQ Total Score	0.627 ****	0.084	−0.138
HAM-A	HAM-A Total Score	0.056	0.171 *	−0.148
HAM-D	HAM-D Total Score	0.140	0.164	−0.095
MRS	MRS Total Score	0.176^*^	0.128	−0.009

PTH: parathyroid hormone; B-TEMPS: Temperament Evaluation of Memphis, Pisa, Paris and San Diego; CTQ: Childhood Trauma Questionnaire; HAM-A: Hamilton Rating Scale for Anxiety; HAM-D: Hamilton Depression Rating Scale; MRS: Mania Rating Scale. * correlation is significant at level of 0.05 (two-tailed correlation). ** correlation is significant at level of 0.01 (two-tailed correlation). *** correlation is significant at level of 0.001 (two-tailed correlation). **** correlation is significant at level of 0.0001 (two-tailed correlation)

**Table 4:** Linear regression models.

Dependent Variable	Independent Variable	B	Standard Error	Beta	*t*	*p*
PTH	Age of Onset	−0.500	0.223	−0.289	−2.238	0.032
	Total Number of Hospitalizations	5.741	2.278	0.160	2.520	0.017
	Aggressive Behaviors	−7.424	4.278	−0.115	−1.735	0.092
	CTQ Total Score	0.904	0.248	1.276	3.637	0.001
	Treatment with Lithium	11.580	4.403	0.179	2.630	0.013
vit d	Age of Onset	0.247	0.201	0.174	1.231	0.224
	Total Number of Hospitalizations	−1.209	1.971	−0.090	−0.614	0.542
	Aggressive Behaviors	3.746	4.082	0.136	0.918	0.363
	CTQ Total Score	−0.072	0.081	−0.129	−0.892	0.376
	Treatment with Lithium	0.530	3.802	−0.020	−0.139	0.890
Calcium	Age of Onset	−0.003	0.007	−0.059	−0.428	0.670
	Total Number of Hospitalizations	−0.064	0.062	−0.143	−1.033	0.306
	Aggressive Behaviors	0.051	0.131	0.055	0.393	0.696
	CTQ Total Score	0.001	0.003	0.040	0.294	0.770
	Treatment with Lithium	0.054	0.123	−0.061	−0.444	0.659

PTH: human parathyroid hormone; CTQ: childhood trauma questionnaire.
